# Quantification of Biomolecular Condensate Volume Reveals Network Swelling and Dissolution Regimes during Phase Transition

**DOI:** 10.1021/acs.biomac.4c01201

**Published:** 2024-12-02

**Authors:** Iris B. A. Smokers, Evan Spruijt

**Affiliations:** Institute for Molecules and Materials, Radboud University, Heyendaalseweg 135, 6523 AJ Nijmegen, The Netherlands

## Abstract

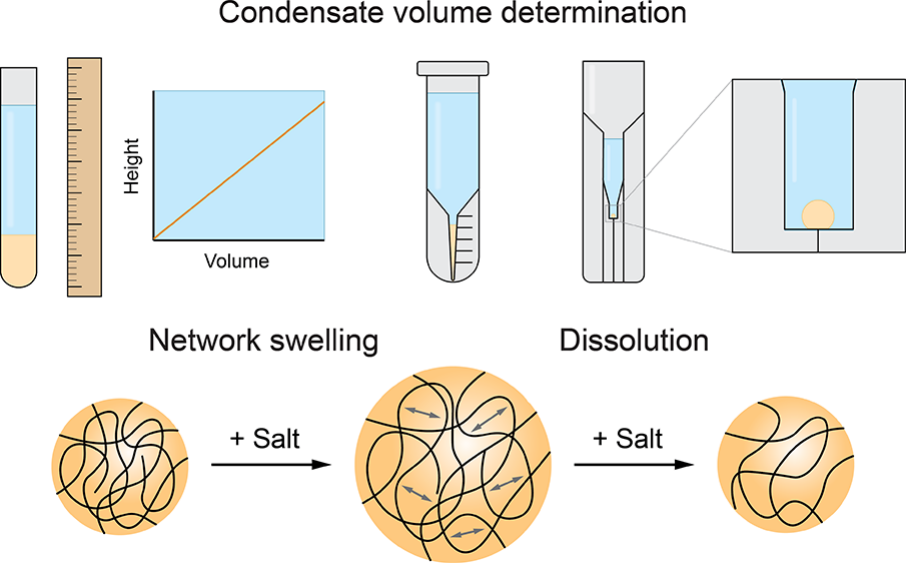

Accurate determination of biomolecular condensate volume reveals that destabilization of condensates can lead to either swelling or shrinking of condensates, giving fundamental insights into the regulation of the volume of cellular condensates. Determination of the volume of biomolecular condensates and coacervate protocells is essential to investigate their precise composition and impact on (bio)chemical reactions that are localized inside the condensates. It is not a straightforward task, as condensates have tiny volumes, are highly viscous, and are prone to wetting. Here, we examine different strategies to determine condensate volume and introduce two new methods, with which condensate volumes of 1 μL or less (volume fraction 0.4%) can be determined with a standard deviation of 0.03 μL. Using these methods, we show that the swelling or shrinking of condensates depends on the degree of physical cross-linking. These observations are supported by Flory–Huggins theory and can have profound effects on condensates in cell biology.

## 1 Introduction

Biomolecular condensates are widely recognized as vital cellular compartments that can localize biomolecules and affect the efficiency of biochemical reactions.^1−4^ Condensates can enhance the activity of certain enzymes^5−10^ and ribozymes^11,12^ and modulate aggregation of prion proteins.^13^ They are also hypothesized to have played a role in the origin of life by concentrating and accelerating prebiotic reactions like the self-replication of genetic information.^14−17^

Condensates are droplets formed by liquid–liquid phase separation (LLPS) of disordered polymers such as proteins, RNA, short peptides, and synthetic polymers, sometimes together with small charged molecules such as ATP.^1,4,18−21^ When condensates are mimicked in vitro using simpler, sometimes nonbiological materials, they are often called coacervates. The liquid–liquid phase separation underlying the formation of both condensates and coacervates gives rise to droplets of a solute-dense phase (the condensate or coacervate) dispersed in a solute-depleted dilute phase (the supernatant). Inside the condensate phase, not only the condensate-forming components are enriched but also guest molecules and ions can be locally concentrated, including proteins,^5^ RNA,^22−24^ and small molecules such as metabolites,^19^ amino acids,^25^ and short peptides.^23,24^ Reactions between guest molecules can be accelerated in condensates due to this increased local concentration and due to the distinctly different local environment.^17,26^ To unravel the composition of condensates and the effects of condensates on (bio)chemical reactions, it is essential to quantify the local concentrations of guest molecules. Moreover, it has recently been shown that the ratio of condensate phase to dilute phase volume can have a significant and nonmonotonic influence on the overall rates and yields of chemical reactions.^26−29^

The most frequently used method to measure the local concentration inside condensates is confocal microscopy. The concentration derived from fluorescence intensities can, however, deviate dramatically from the actual concentration, for instance, because of differences in the quantum yield of the fluorophores between the condensate and dilute phase.^15,30^ Moreover, this method cannot be used for small molecules, such as ATP and many enzyme substrates, because attaching a fluorescent label significantly alters the size and physicochemical properties of such molecules.

In these cases, concentrations in the condensate and dilute phase can be measured by NMR, HPLC, or UV–vis spectroscopy after centrifugation and separation of the phases and dissolution of the condensate phase. This approach requires that the volume of the condensate phase is known. However, quantifying condensate volumes is fraught with difficulties.

For most biological- and peptide-based condensates, the condensate volume fraction is small, typically in the range of 0.01–1 v/v %.^22^ In addition, they can be difficult to handle due to high viscosity, low surface tension, and tendency to wet many types of surfaces. These properties also trouble the volume determination of synthetic condensates, even though larger volume fractions of 20–90 v/v % have been achieved for these condensates.^21,31^ Small errors have an enormous effect on calculated concentrations, and preparation of larger samples or higher concentrations is often not feasible. Development of accurate methods to determine small condensate volumes is therefore crucial.

In this paper, we examine the accuracy of several methods used in recent literature for estimating condensate volume, and we add two new methods. We focus on methods that are simple and can be carried out with standard laboratory equipment. We determine the accuracy and precision of these methods and discuss advantages and disadvantages of each method for different applications. We then use these methods to analyze how the condensate volume changes as they approach a critical point, for example, by the addition of salt, for different types of condensates. Interestingly, condensate dissolution as the critical point is approached has two distinct regimes: expansion of the molecular network in the condensate leading to an increase in volume and dissolution due to the release of condensate components from the droplets, leading to a rapid decrease in volume. Whether both regimes are observed depends on the relative sizes of the molecules forming the condensate network. Our findings have implications for condensate volume regulation and rates of biochemical reactions in living cells, where many condensates may exist close to their critical points to allow the cell to actively control their formation and dissolution.^32,33^ Slight variations in environmental conditions or protein–protein interactions may lead to a drastic reduction in the volume of some condensates but swelling of others and can have profound effects on local concentrations of RNA, transcription factors, chaperones, and other components in cellular condensates.

## 2 Experimental Section

### 2.1 Materials

All chemicals and reagents were used as received from commercial suppliers unless stated otherwise. We used Milli-Q water (i.e., ultrapure deionized water, 18.2 MΩ cm) from Millipore Corporation. The following compounds were purchased from Sigma-Aldrich: protamine chloride from salmon (grade V, histone free), adenosine 5′-triphosphate disodium salt hydrate, poly(acrylic acid) sodium salt (PAA, 15 kDa, 35 wt % solution in H_2_O), poly(diallyldimethylammonium chloride) (PDDA, 200–350 kDa, 20 wt % solution in H_2_O), and sodium chloride. 1.0 M hydrochloric acid and 1.0 M sodium hydroxide were purchased from Fisher Scientific. Tris(hydroxymethyl)-aminomethane was purchased from Merck Millipore. PLL-*g*[3.5]-PEG was purchased from SuSoS. Poly[2-(methacryloyloxy)-ethyl]trimethylammonium chloride (PMETAC, *N* = 170, PDI = 1.3) and 5% fluorescein-labeled poly(3-sulfopropyl methacrylate) potassium salt (Fl-PSPMA, *N* = 210, PDI = 1.3) were synthesized previously by atom transfer radical polymerization (ATRP) following the procedure from Spruijt et al.^34,35^ For the 5% fluorescein-labeled PSPMA, 5% fluorescein methacrylate was copolymerized with the sulfopropyl methacrylate.

### 2.2 Condensate Preparation Protamine/ATP

To ensure charge neutrality of the condensates, we selected the ratio of protamine:ATP that gave the most stable condensates (i.e., highest critical salt concentration (CSC)). A composition of 1 mM protamine with 25 mM ATP gave the highest CSC: 585 mM.

Condensate emulsions of 1 mM protamine (molecule-based) and 25 mM ATP (molecule-based) in 50 mM tris pH 8.5 were prepared using stock solutions of 4 mM protamine chloride (grade V, Histone free) in 50 mM tris pH 8.5, 100 mM adenosine 5′-triphosphate disodium salt hydrate (ATP) in 50 mM tris pH 8.5, and 2 M sodium chloride in 50 mM tris pH 8.5. All stock solutions were corrected back to pH 8.5 using 1 M NaOH. For a 1 mL condensate sample, 250 μL of 4 mM protamine chloride was added to 500 μL of 50 mM tris pH 8.5 and the solution was pipetted up and down several times. Subsequently, 250 μL of 100 mM ATP was added, upon which the solution became turbid. The emulsion was mixed either by pipetting up and down several times (for the cell counting tubes and sessile droplet method) or by vortexing for a few seconds and inverting the tube 3× (for the mass-based method and calibrated height measurement).

For the salt-induced dissolution measurements, condensate emulsions of 1 mM protamine and 25 mM ATP were prepared in a similar way, but in 100 mM tris pH 8.5, in the presence of the appropriate concentration of sodium chloride.

The lab temperature was recorded for all experiments and was consistently between 19.5 and 21.0 °C but never varied more than 0.5 °C during a single experiment.

### 2.3 Condensate Preparation PDDA/PAA

Charge-neutral condensate emulsions of 65 mM (monomer-based) poly(diallyldimethylammonium chloride) (PDDA, 200–350 kDa) and 65 mM (monomer-based) poly(acrylic acid) sodium salt (PAA, 15 kDa) in 100 mM tris pH 8.5 were prepared using stock solutions of 260 mM PDDA in 100 mM tris pH 8.5, 260 mM PAA in 100 mM tris pH 8.5, and 2 M sodium chloride in 100 mM tris pH 8.5. All stock solutions were corrected back to pH 8.5 using 1 M NaOH and 1 M HCl. For a 1 mL condensate sample, the required volume of 2 M sodium chloride and 250 μL of 260 mM PDDA were added to the 100 mM tris pH 8.5, and the solution was mixed by vortexing for a few seconds. Subsequently, 250 μL of 260 mM PAA was added, upon which the solution became turbid. The emulsion was mixed by vortexing for a few seconds and inverting the tube at least 3×.

Charge-neutral condensate emulsions of 150 mM (monomer-based) poly(diallyldimethylammonium chloride) (PDDA, 200–350 kDa) and 150 mM (monomer-based) poly(acrylic acid) sodium salt (PAA, 15 kDa) in 100 mM tris pH 8.5 were prepared in a similar fashion using stock solutions of 600 mM PDDA in 100 mM tris pH 8.5, 500 mM PAA in 100 mM tris pH 8.5, and 2 M sodium chloride in 100 mM tris pH 8.5.

### 2.4 Volume Determination by Mass

To determine the mass fraction of the condensate phase, 2 mL condensate samples were prepared in 2 mL Eppendorf tubes, for which the empty weight was determined in advance, following the preparation method above. The condensate emulsion was mixed by pipetting up and down several times and vortexing for a few seconds, after which the full tube was weighed and the sample was left to equilibrate for 20 min. It was subsequently centrifuged for 30 min at 3100 RCF and 20 °C. After centrifugation, the supernatant was clear, and the condensate was collected as a slightly opaque liquid at the bottom. Most of the dilute phase was removed by a micropipette, making sure that the pipette tip did not touch the condensate phase. The last droplets of the dilute phase were removed with filter paper, resulting in an isolated condensate phase, and the tube with the condensate phase was weighed again. The process is depicted in Supporting Figure 1. All experiments were carried out in triplicate. The mass fraction was calculated according to eq 1
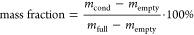
1To calculate the volume fraction, the density of the condensate phase was needed. To determine this, four cell counting tube (CCT) samples were prepared following the procedure described in Section 2.6. The empty tubes were weighed before sample preparation, and after preparation and centrifugation, the dilute phase was removed using a micropipette and syringe with a narrow needle, after which the remaining dilute phase was removed with filter paper. The samples were centrifuged for another 10 min at 3100 RCF and 20 °C to ensure that the condensate interface was not disturbed by the separation of the phases. After the second centrifugation step, the condensate volume was read out, and the sample was weighed to determine the condensate mass. The condensate density was calculated using eq 2
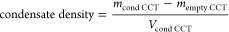
2For the condensates made of 1 mM protamine with 25 mM ATP in 50 mM tris pH 8.5, the condensate density was calculated to be 1460 ± 31 mg/mL. The density of the dilute phase was determined by weighing a known volume of isolated dilute phase and was calculated to be 997 mg/mL, almost exactly equal to the water density at 25 °C (997 mg/mL). The volume fraction of the mass-based samples was then calculated according to eqs 3 and 4
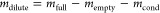
3
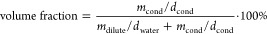
4

### 2.5 Calibrated Height Measurement

The calibrated height samples were prepared in borosilicate glass test tubes with an outer diameter of 8 mm (wall thickness 0.8–1.0 mm, DWK Life Sciences). 1 mL condensate samples were prepared following the method above, after which the emulsion was mixed by vortexing for a few seconds and inverting the tube at least 3×. The samples were left to equilibrate at room temperature for 30 min, after which they were centrifuged for 30 min at 3095 RCF and 20 °C in custom 3D-printed centrifuge holders. The condensate volume and total sample volume were determined by measuring the length from the bottom of the tube to the interface/meniscus with a ruler (Supporting Figure 2). This was done directly after centrifugation and after the samples had been equilibrated at room temperature for 30–40 days. For some PDDA/PAA samples close to the critical salt concentration, the interface was almost invisible. A short heat shock (5–10 s at 35 °C) was used to visualize the interface. The measured lengths were fit to the calibration curve in Supporting Figure 3 to determine the volume. The calibration curve was prepared using known volumes of Milli-Q water, which were centrifuged for 1 min at 3095 RCF and 20 °C.

### 2.6 Volume Determination by Cell Counting Tube

PCV cell counting tubes (capillary graduations only, no cap, Sigma-Aldrich) were used directly without surface modification. 1 mL condensate samples were prepared directly in cell counting tubes following the preparation method above, after which the emulsion was mixed by pipetting up and down several times. The samples were centrifuged for 30 min at 3100 RCF and 20 °C directly after preparation. The condensate volume was read out from the graduations (Figure 2a and Supporting Figure 5). All experiments were carried out in triplicate.

It is important to note that preparation of the sample in a separate Eppendorf and subsequent transfer to the cell counting tube and/or waiting with centrifugation for more than 5 min after condensate preparation resulted in a significant reduction of the observed condensate volume.

The accuracy of the cell counting tube read-out was checked by adding 1, 2, 3, or 4 μL MilliQ-water to cell counting tubes and centrifuging them for 30 s at 3100 RCF and 20 °C. A shorter centrifugation time was used to avoid evaporation of the small volume of water. Supporting Figure 6 shows that the cell counting tubes provide a reliable read-out of these volumes.

### 2.7 Sessile Droplet Method

This procedure was adapted from Holland et al.^36^ Disposable cuvettes (BRAND UV cuvette micro, center H 15 mm, volume 70–550 μL, pack of 100 ea) were modified using pLL-*g*-PEG using the following procedure: the cuvettes were cleaned using a plasma cleaner, after which they were filled with 100–300 μL (at least as much as the sample volume) of 0.01 mg/mL pLL-*g*-PEG dissolved in 10 mM HEPES pH 7.4. They were incubated for 24 h at room temperature and subsequently washed three times with water and dried with pressurized air.

100 μL condensate samples were prepared directly in the cuvette following the preparation method above, after which the cuvette was sealed with parafilm and the sample was left to equilibrate for 20 min. The cuvettes were centrifuged for 30 min at 3095 RCF and 20 °C, after which the condensate phase was clearly visible as a spherical droplet at the bottom of the cuvette chamber, which could roll under the influence of gravity if the cuvette was held at an angle of 10°. In some cases, several droplets had formed, which could be combined into a single droplet by rolling them toward each other and letting them fuse. In a few cases, the droplet was strongly stuck to the wall of the cuvette, which was attributed to bad surface modification, and in these cases, the sample was prepared again.

To determine the droplet volume, the droplet was positioned close to the front of the cuvette at a position where it did not touch any of the walls of the cuvette. A First Ten Ångströms FTA1000 Drop Shape Instrument B Frame System goniometer equipped with an Artray Artcam 130MI-BW camera was used to take a picture in which both the full droplet and the walls of the cuvette chamber were in focus.

Condensate droplet volume was calculated from the obtained image using ImageJ. Using the known dimensions of the cuvette chamber (2 mm width), the dimensions of the droplet could be calculated using the spherical cap or elliptical cap method (Supporting Information Section 3.5).

### 2.8 Volume Determination as a Function of Salt

PDDA/PAA (200–350 and 15 kDa, respectively) at monomer concentrations of 65 and 150 and 50 mM PMETAC/(5% fluorescein-)PSPMA condensates were prepared as detailed above and in Supporting Information Section 2.1. The change in condensate volume as a function of sodium chloride concentration was determined using the calibrated height measurement method. 1 mM protamine/25 mM ATP condensates in 100 mM tris pH 8.5 were prepared as detailed above, and their change in condensate formation as a function of salt was determined using cell counting tubes. The total concentration of NaCl is calculated by adding the concentration of added NaCl and the concentration of polymer counterions in the sample. For protamine/ATP, there is a higher concentration of Na^+^ (90 mM) than Cl^–^ (23 mM). The concentration of Cl^–^ was taken as the counterion concentration.

### 2.9 Flory–Huggins Prediction of Condensate Volume as a Function of χ

Flory–Huggins theory was used to predict the change in condensate volume for a two-component (polymer–solvent) mixture as a function of the interaction parameter χ. Analytical approximations of near-critical binodals from Van Leuken et al.^37^ were used
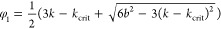
5
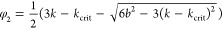
6
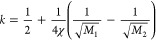
7
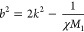
8where φ_1_ and φ_2_ are the volume fractions of the polymer in phase 1 (the condensate) and phase 2 (the dilute phase), respectively, *M*_1_ is the length of the polymer, and *M*_2_ = 1 corresponds to the solvent. The condensate volume fraction was calculated according to
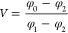
9

## 3 Results and Discussion

### 3.1 Comparison of Volume Measurement Methods

**Figure 1:**
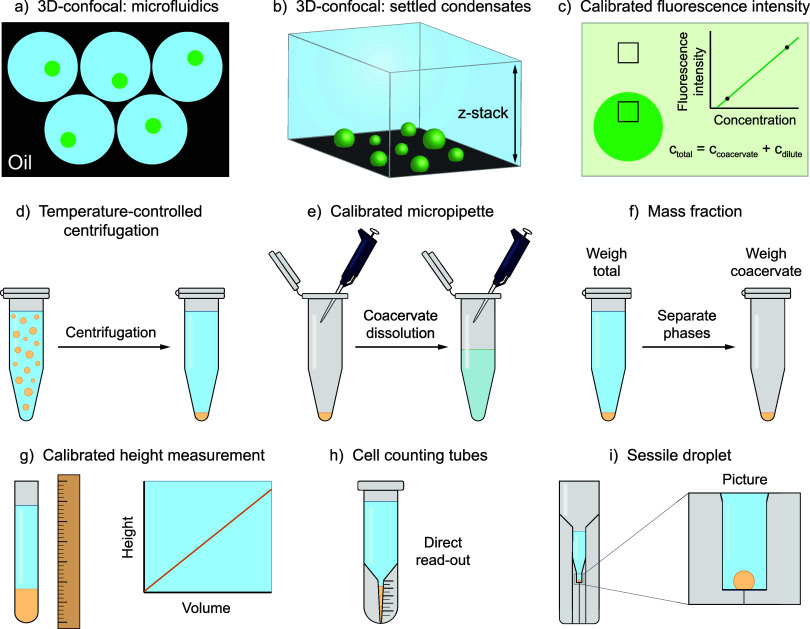
Schematic representations of methods to determine the condensate volume fraction. (a–c) 2D/3D confocal-based methods: (a) volume determination of a single condensate droplet in water-in-oil droplets in a microfluidic setup; (b) volume determination using a large *z*-stack to capture the settled condensate droplets and total dilute phase; (c) volume determination using calibrated fluorescence intensities of guest molecules in the condensates and using the conservation of mass. (d–i) Methods that require (d) centrifugation of the sample to get a single macroscopic condensate phase: (e) measuring condensate volume using a calibrated micropipette after manual separation of the phases; (f) weighing the condensate phase and total sample mass to determine the mass fraction after manual separation of the phases; (g) volume determination by calibrated height measurement; (h) volume determination by cell counting tubes; and (i) volume determination via the sessile droplet method.

To determine the condensate volume, several methods have recently been used in the literature (Figure 1 and Table 1). In vivo, the condensate volume is typically determined using 3D confocal microscopy. Since condensates are suspended in the cell, a *z*-stack can be made to determine the droplet volume, but determination of the volume fraction is more challenging, as the total system volume is difficult to determine.^38^ In vitro, 3D confocal can be used to determine the volume fraction for single condensate droplets confined to water-in-oil droplets or vesicles prepared, for example, by microfluidics (Figure 1a),^39−41^ since both the condensate volume and total sample volume can be determined readily (see Supporting Information Section 1.1). 3D confocal can also be used for condensates settled on passivated microscopy slides. In this case, a large field of view should be imaged to get an estimate of the condensate volume fraction to the total sample volume (Figure 1b and Supporting Information Section 1.2).^7^ A disadvantage of this method is that such large *z*-stacks are prone to optical aberrations. Moreover, many condensates are found to adhere to cellular interfaces, including membranes, and their shape can strongly deviate from spherical.^42−45^ This makes determining their volume using confocal microscopy nontrivial.

**Table 1:** Overview of the Different Methods for Condensate Volume Determination^a^

method	sample volume	accuracy	precision	advantages	disadvantages	main source of error	required instruments
3D confocal: microfluidics	20–50 pL^b^	high	high	in situ, no centrifugation required, high throughput	requires a microfluidic setup and suitable encapsulation method, proteins may denature when in contact with fluorinated oils or surfactants	non-equal mixing ratios, contact with fluorinated oils or surfactants	confocal microscope, microfluidic setup
3D confocal: settled condensates	10–100 μL	low	intermediate	in situ, no centrifugation	requires fluorescent label and surface modification, large-scale *z*-stack, prone to optical aberrations	optical aberrations	confocal microscope
calibrated fluorescence intensity	10–100 μL	intermediate	high	in situ, no centrifugation	requires fluorescent label and surface modification, differences in quantum yield between condensate and dilute phase	differences in quantum yield, low signal in dilute phase	confocal microscope
calibrated micropipette	100 μL^c^–10 mL	low	low		sticky condensate, manual separation of phases	condensate sticking to pipette	temperature-controlled centrifuge, calibrated micropipette
mass fraction	500 μL–10 mL	low to intermediate, depends on volume	low to intermediate	easily combined with determining water content	large sample volume, manual separation of phases	manual separation of phases not perfect	temperature-controlled centrifuge, balance
calibrated height measurement	100 μL–10 mL	intermediate	intermediate	measurement of dilute phase volume^d^	visual inspection, relatively large sample volume	visual read-out difficult	temperature-controlled centrifuge
cell counting tubes	500 μL–1 mL	high	high	easy read-out	relatively large sample volume	not centrifuging directly after preparation	temperature-controlled centrifuge
sessile droplet	50–200 μL	intermediate	intermediate	lowest required sample volume	requires surface modification	poor surface modification	temperature-controlled centrifuge, camera

a Required sample volume is estimated for a 0.1–1 v/v % condensate volume fraction.

b Per water-in-oil droplet. A larger total volume will be needed to create the droplets in the microfluidic setup.

c Only for the dissolved condensate phase.

d When prepared in an NMR tube or other cylindrical tubes.

Alternatively, regular confocal fluorescence microscopy can be used to determine the condensate volume using calibrated fluorescence intensities of guest molecules and conservation of mass (Figure 1c and Supporting Information Section 1.3).^7^ For this method, the total mass of guest molecules must be known, and a calibration curve for fluorescence intensities has to be prepared. Even though the microfluidic and calibrated fluorescence intensity methods work in situ for (very) small sample volumes (20 pL–100 μL), these (confocal) fluorescence microscopy-based methods have the disadvantage that fluorescent labeling is required, and the calibrated fluorescence intensity method may suffer from the same limitations concerning quantum yields, as discussed above.

There are also several label-free methods. All of these methods require centrifugation of the condensate emulsion at a controlled temperature to induce coalescence of the condensate droplets and collect them in a macroscopic condensate phase, usually at the bottom of the tube with the dilute phase on top (Figure 1d). It should be noted that because centrifugation induces shear, these methods might not be suitable for condensates that are prone to liquid-to-solid transition.^46,47^ When the dilute phase is removed after centrifugation, the amount of condensate phase can be determined either by pipetting or by weighing the condensate phase. In the first case, a calibrated pipette is used to determine the volume of the condensate phase (Figure 1e), either by pipetting the condensate phase directly,^48^ by dissolving the condensate phase and determining the volume of the dissolved condensate phase,^49^ or by determining the volume of the removed dilute phase.^23^ However, the high viscosity and sticky nature of many condensates, volume changes upon mixing, and difficulties to dissolve all condensate material can all lead to large errors in the volume. In addition, large sample volumes are typically required (100 μL–10 mL). For condensates made of 1 mM protamine and 25 mM ATP in 50 mM tris buffer pH 8.5 that were used to benchmark other methods in this paper, the calibrated pipetting method did not work: the pure condensate phase was too viscous to pipette, and the dissolved condensate phase adhered so strongly to the outside of the pipette tips that the measured volume of the dissolved condensate phase was less than the volume added to dissolve it.

An alternative to pipetting is weighing the total sample and the isolated condensate phase and determining the mass fraction of the condensate phase (Figure 1f).^50^ This method is suitable also for condensates with strong surface adhesion and can easily be combined with drying of the condensate phase to determine the condensate water content.^51^ It does, however, also require a large sample volume (500 μL–10 mL for a 0.1–1 v/v % condensate fraction) to be able to accurately separate the phases and weigh the condensate phase.

There are also methods that do not require isolation of the separated phases. The most frequently used, and most straightforward method, is to measure the height or size of the condensate phase in an Eppendorf tube^22,52^ or narrow NMR tube (Figure 1g) and compare with standards of known volumes.^53^ The latter is the preferred choice, as due to the cylindrical shape of the NMR tubes, the condensate volume can be quantified with a ruler, which is more reliable than visual estimation of the droplet size in an Eppendorf tube.^52^ Especially for large condensate volume fractions (>5 v/v %) the use of narrow tubes is a very suitable method for volume determination.

**Figure 2:**
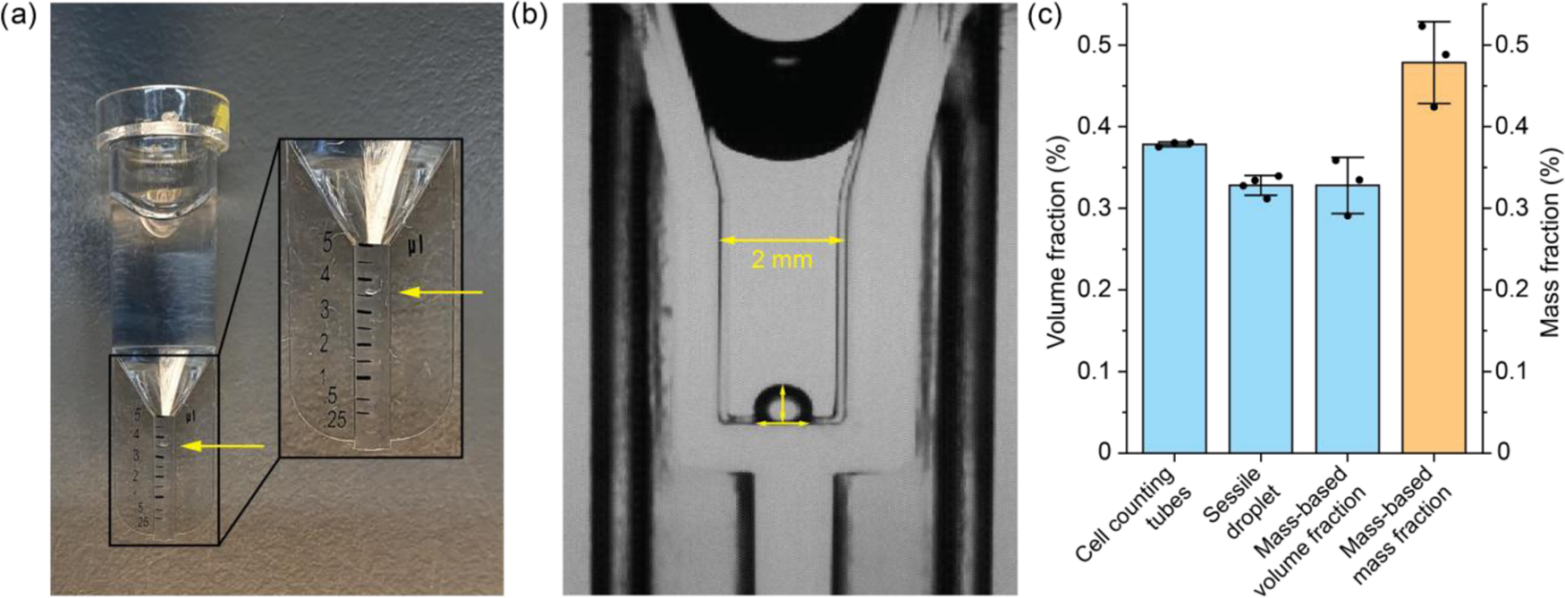
Comparison of different volume determination methods for condensate samples of 1 mM protamine and 25 mM ATP in 50 mM tris buffer, pH 8.5. (a) Image of a cell counting tube; the yellow arrow indicates the interface. (b) Obtained image for the sessile droplet method in a surface-modified cuvette. The droplet volume can be calculated using the known width of the cuvette chamber and by approximating the droplet shape as a spherical or elliptical cap. (c) Comparison of the mean volume fraction determined by cell counting tubes, sessile droplet method, and mass-based method. For the mass-based method, both the mass fraction and volume fraction (calculated using the condensate density) are shown. Error bars indicate the standard deviation of a triplicate measurement.

Inspired by the calibrated height measurement method, we present cell counting tubes as an improved version for small condensate volume fractions (0.05–1 v/v %) that allows direct read-out of the condensate phase volume after centrifugation (Figure 1h). These tubes have a narrow graduated capillary at the bottom of a 1 mL vial (Figures 1h and 2a), which allows for accurate read-out of microliter volumes of condensate phase, ideal for condensate systems with volume fractions in the 0.1–1 v/v % range. It does require a relatively large sample volume of 500 μL–1 mL.

For condensates with lower total sample volume (50–200 μL), we present a method adapted from Holland et al. using image-based analysis of a single sessile condensate droplet (Figures 1i and 2b). The method was developed for surface tension measurements of small condensate samples but also allows determination of condensate volume.^36^ A condensate sample is added to a PLL-*g*-PEG passivated UV-polymer cuvette and gently centrifuged, resulting in the formation of a single condensate phase droplet that does not wet the cuvette bottom or side walls and that can slide across the cuvette bottom surface when tilted: for tilting angles larger than 10°, the droplet would roll under the influence of gravity. A goniometer or microscope can be used to take a picture of the droplet, from which the volume can be calculated by approximating the droplet shape as a spherical or elliptical cap (see Supporting Information Section 3.5). A requirement of this method is the use of an appropriate surface modification that prevents wetting by the condensate and allows for the formation of a sessile droplet. For different types of condensates, different surface modifications might have to be used.

After having established the main advantages and limitations of the different methods to determine condensate volume and having identified the most promising methods for low-volume, viscous condensate samples, we decided to make a quantitative comparison of the methods that can be carried out without the use of microscopy and benchmark their accuracy and precision. For the mass-based method, the cell counting tube method, and the sessile droplet method (Figure 2a,b), we determined the condensate volume fraction of a sample containing 1 mM protamine and 25 mM ATP in 50 mM tris buffer pH 8.5 in triplicate (Figure 2c). The calibrated micropipette method was left out of the comparison because it did not work for this condensate system (as detailed above). The calibrated height measurement only works for larger condensate volume fractions and was therefore only used for the synthetic polymer condensates in Section 3.2.

When we compare the standard deviations of the three different methods, we can clearly see that the cell counting tube method is the most precise (s.d. = 0.003%), followed by the sessile droplet method (s.d. = 0.015%) and the mass-based method (s.d. = 0.020%). For the mass-based method, the mass fraction could only be converted to a volume fraction by determining the condensate density (see Section 2.4).

The cell counting tube method consistently gives a higher condensate volume fraction than the sessile droplet- and mass-based methods. Considering that any step in the volume determination procedure can lead to loss or underestimation of the condensate phase volume—e.g., loss during manual separation of the phases or sticking of condensate phase to the side of the tube during centrifugation—we see no reason to expect a volume fraction overestimation, and therefore we expect the highest condensate volume fraction to be the most accurate. Based on these results, cell counting tubes are advised for measuring low condensate volume fractions (0.1–1 v/v %) in a sufficiently large total sample volume, as this method is the easiest to use, the most accurate, and the most precise. For (biological) condensates where the total sample volume is limited, the sessile droplet method may be a suitable alternative, as it has decent accuracy and precision and requires only a 50–200 μL sample. Additionally, we envision that these techniques can be used for other LLPS systems such as oil-in-water droplets or aqueous two-phase systems.

### 3.2 Influence of Salt on Condensate Volume

**Figure 3:**
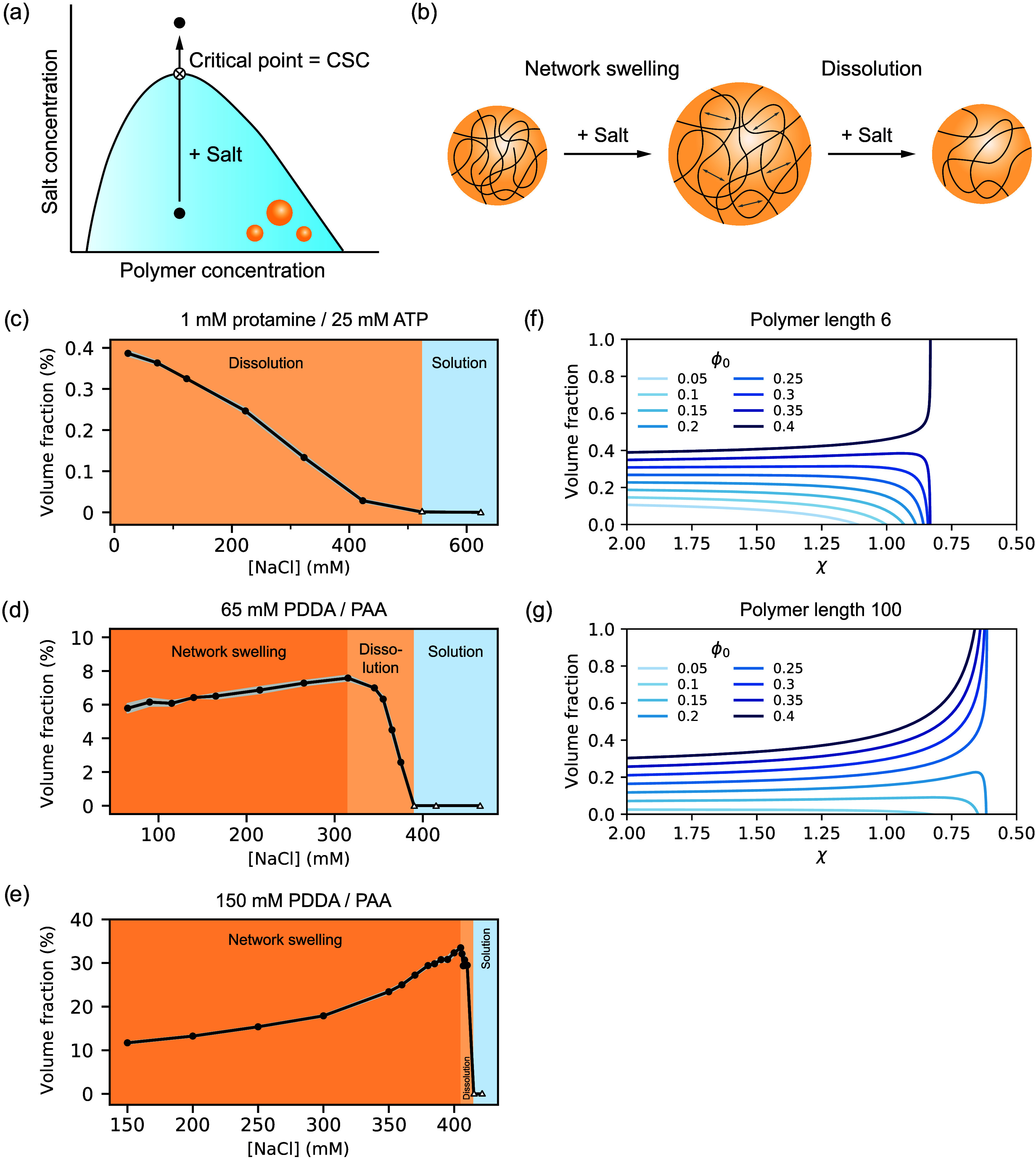
Evaluation of the condensate volume fraction as a function of salt concentration shows two distinct regimes: network swelling and dissolution. (a) Phase diagram for a charge-based condensate, which is destabilized by salt due to weakening of charge–charge interactions and lowering of the entropic gain of counterion release upon phase separation. At the critical point/critical salt concentration (CSC), the composition of the condensate phase is equal to the dilute phase composition and the condensates dissolve. (b) Schematic illustration of the network swelling regime and the dissolution regime. (c) Changes in volume fraction as a function of total sodium chloride concentration (added salt + counterions of condensate components) for 1 mM protamine/25 mM ATP condensates in 100 mM tris pH 8.5, measured directly after preparation. For panels (c–e), empty triangles indicate the samples for which no phase separation was observed. Error bars are shown as shaded regions and depict the standard deviation of measurements in triplicate. (d) Changes in volume fraction as a function of total sodium chloride concentration for 65 mM PDDA/PAA condensates in 100 mM tris pH 8.5, measured directly after preparation. (e) Change in volume fraction as a function of total sodium chloride concentration for 150 mM PDDA/PAA condensates in 100 mM tris pH 8.5 measured after allowing the samples to equilibrate for 1 month. (f, g) Predictions of the condensate volume fraction for a two-component (polymer–solvent) mixture according to Flory–Huggins theory as a function of the interaction parameter χ for polymers of different lengths but same χ_crit_ (=0.4): (f) polymer length 6, (g) polymer length 100. Network swelling is observed more readily for the longer polymer.

Measuring the volume of condensates is essential for quantitative analysis of partitioning, local concentration of guest molecules, and reaction rates in the condensate phase. However, analysis of the condensate volume itself can also provide new and fundamental insights into liquid–liquid phase separation. To illustrate this point, we investigated the effect of addition of salt on the condensate volume fraction. Addition of salt lowers both the enthalpic and entropic driving force for condensate formation, as it weakens electrostatic interactions between oppositely charged components by screening the charges, and it lowers the gain in entropy by counterion release from the dissolved polymers upon phase separation. Beyond a critical point (the critical salt concentration, CSC), addition of salt dissolves the condensates (Figure 3a). However, how the condensate volume changes toward the critical point is not trivial and may have implications for condensate volume regulation in cells, where they are believed to exist close to their critical points to allow the cell to actively control their formation and dissolution.^32,33^

We investigated three condensate systems, two made with the long synthetic polymers PDDA/PAA (200–350 and 15 kDa, respectively, at monomer concentrations of 65 and 150 mM for both polymers) and PMETAC/PSPMA (34 and 52 kDa, respectively, at monomer concentrations of 50 mM) and one short peptide-based system made with 1 mM protamine and 25 mM ATP. For the long synthetic polymers, we assumed charge neutrality for equal monomer concentrations. For protamine/ATP, we selected the ratio that gave the highest CSC (i.e., highest condensate stability). We prepared condensate samples with different concentrations of sodium chloride up to the CSC and measured the volumes either with cell counting tubes (for protamine/ATP) or using a calibrated height measurement in narrow test tubes (for PDDA/PAA), as this is the more suitable method for higher volume fractions of condensate phase.

For the protamine/ATP condensates, we observe a gradual dissolution of condensates: the condensate volume fraction starts at 0.39% and continuously decreases upon addition of salt, until it reaches zero at the CSC at 520 mM (total) salt (Figure 3c and Supporting Figure 9). A similar trend was recently observed by Chen et al. for polylysine/ATP condensates in microfluidic droplets.^41^ For 65 mM PDDA/PAA, the condensate volume fraction ultimately decreases to zero for high salt concentrations (Figure 3d and Supporting Figure 10). However, unlike for protamine/ATP condensates, we initially observe a significant increase in condensate volume fraction (5.5–7.5%) from 65 mM total salt up to 315 mM, which we attribute to swelling of the polymer network (Figure 3b). Beyond 315 mM total salt, the condensate volume fraction decreases sharply (7.5 to 0%) from 315 to 390 mM NaCl. A similar trend is observed for more gel-like condensates of 50 mM PMETAC/(5% fluorescein)-PSPMA, which become more liquid-like at the onset of the sharp decrease in volume fraction (Supporting Figure 11).

The network swelling effect is dramatically amplified for higher concentrations of the condensate-forming components (Figure 3e and Supporting Figure 12). Upon addition of salt, the volume fraction of PDDA/PAA condensates at 150 mM monomer concentrations for both polymers increases by a factor of 3 (from 11.7 to 33.5%) during the network swelling regime, while the dissolution regime is more narrow (from 405 to 415 mM) and the corresponding decrease in volume fraction is very dramatic, indicating that the location in the phase diagram (i.e., overall polymer concentration) determines not only the condensate volume fraction but also the degree to which this fraction increases by network swelling and the sharpness of the dissolution regime.

Interestingly, such a transition between swelling and dissolution and the varying degrees of swelling are in qualitative agreement with classical mean-field Flory–Huggins theory that is commonly used to describe LLPS (Figure 3f,g).^37,41^ We used analytical approximations to near-critical binodals from Van Leuken et al.^37^ for a two-component (polymer–solvent) mixture to model the change in condensate volume according to Flory–Huggins theory (Section 2.9), as a function of the interaction parameter χ for different polymer lengths. The interaction parameter χ is a measure of the interaction strength between the polymer and solvent relative to their self-interaction. High positive values reflect more unfavorable interactions between the polymer and the solvent. Although complex coacervates, including the systems studied in Figure 3, are multicomponent systems (typically containing a polycation, polyanion, buffer, monovalent salt, and solvent), their phase behavior at 1:1 charge ratio can be mapped onto Flory–Huggins theory for a polymer in solution, as was shown earlier,^54−58^ by defining an effective interaction parameter, which is a function of the polymer charge density, temperature, salt concentration, and hydrophobicity.^58^ A decrease in χ represents a decrease in the driving force for coacervate formation, which could be obtained by changes in these parameters. Interestingly, this simple Flory–Huggins model predicts the same behavior of network swelling and dissolution as observed experimentally for complex coacervates. Whether both regimes are observed depends on both the total polymer volume fraction φ_0_, and χ_crit_, the value of the Flory–Huggins interaction parameter at the critical point. In our model, we kept χ_crit_ constant at an arbitrary fixed value to investigate the effect of changes in polymer length and condensate volume fraction. Similar to our observations for higher polymer concentrations, the theory predicts that for higher total polymer volume fractions φ_0_, the increase in volume fraction due to network swelling is larger and the dissolution regime becomes narrower. This holds for both short (length *M*_1_ = 6; Figure 3g) and long polymers (length *M*_1_ = 100; Figure 3h), although for the shorter polymer a significantly larger φ_0_, i.e., higher polymer concentration, is required to observe a network swelling regime, matching our observation that the protamine/ATP condensates did not go through a network swelling regime, while the PDDA/PAA did.

The theory does predict, however, that for higher concentrations the network swelling regime should also be observed for protamine/ATP. Interestingly, at very large φ_0_, the dissolution regime first becomes very narrow and disappears for even larger φ_0_, where the condensate phase takes over the total sample volume when approaching the critical point. At these high initial polymer volume fractions, the composition of the dissolved uniform system is likely closer to the composition of the condensate phase than to the composition of the dilute phase, causing the condensate phase to take over the total sample volume when approaching the critical point. Experimentally, obtaining such high polymer concentrations is likely only possible for long synthetic polymers, such as the PDDA/PSS condensates by Wang and Schlenoff (Supporting Figure 13),^21^ where it becomes challenging to determine whether the condensate phase dissolves into the dilute phase, or vice versa, because the change in volume fraction close to the critical point becomes extremely sharp.

### 3.3 Swelling and Dissolution Mechanisms

Our results indicate that condensates can have distinct mechanisms of volume adaptation as the driving force for phase separation changes and they approach their critical points, depending on the nature of the condensate components. For small-molecule-based condensates, only a dissolution regime is observed, where condensate components are released to the dilute phase leading to a continuous decrease in condensate volume fraction. For condensates made from long polymeric components, however, the dissolution regime was preceded by a network swelling regime, where the strongly interconnected polymer network stretches due to interaction with salt ions until it breaks apart and transitions to the dissolution regime. For high polymer concentrations, the dissolution regime can become so narrow that it is not visible anymore, and Flory–Huggins theory predicts that the condensate phase can even take over the total sample volume for very high concentrations.

Condensates are believed to be percolated network fluids,^59^ where the polymers create a network that spans the entire droplet volume. The network is formed by a combination of physical cross-links (e.g., ion pairs or pairs of interacting aromatic stickers) and entanglements for sufficiently long polymers. The network strength is determined by both the strength of individual cross-links between polymers and the total connectivity in the network.^60,61^ The strength of individual polymer–polymer interactions is reflected in the critical point (e.g., critical salt concentration (CSC) or critical temperature) of condensates.^59^ Interestingly, the protamine/ATP condensates have a higher CSC (520 mM NaCl) than the PDDA/PAA condensates we used (390 mM NaCl for 65 mM polymer and 415 mM NaCl for 150 mM polymer, respectively), which may be explained by previous observations in literature that the arginines in protamine form stronger ion pairs with phosphates and carboxylic acids than the quarternary ammoniums of PDDA, which are known to form relatively weak ion pairs in condensates.^52,62^ We can therefore conclude that the main determinant of the mechanism of dissolution is not the strength of individual cross-links, but rather the polymer length and concentration and the corresponding total degree of cross-linking of the polymers. Studying both the CSC and the mechanism of dissolution therefore allows us to discriminate between these two factors.

The cross-links in the network can be weakened by changing solution conditions or temperature, and the probability of a release of a single cross-link can be approximated as e^–Δ*G*/*kT*^, where Δ*G* is the energy of sticker binding or ion pairing. In the condensate, each component is connected to the network by an average number of cross-links that increases with increasing length and sticker or charge density. The probability of releasing a polymeric chain from the condensate decreases exponentially with increasing chain length (e^–*N*Δ*G*/*kT*^ for polymer length *N*). Therefore, small molecules, such as ATP, may be readily released from a condensate, while long polymeric components are unlikely to be released, except very close to the critical point where Δ*G* becomes very small. At the same time, as we approach the critical point, the relative solvent quality for the condensate components improves: above the critical point, the condensate components are in a good solvent. This causes swelling of the polymer network and an increase in the condensate volume, as was indeed observed for condensates formed by long polymers. However, for small-molecule-based condensates, or condensates with only few stickers at moderate concentrations, this swelling is completely suppressed by the simultaneous release of the condensate components, resulting in a net decrease in condensate volume. The degree of swelling is larger for condensates that are closer in composition to the average composition of the mixture: they must take up a larger amount of the coexisting phase with its dissolved polymers to reach the same equilibrium composition.

Following our predictions by Flory–Huggins theory, we expect the response in salt-induced dissolution to be translatable to other means of condensate destabilization, such as changes in temperature, pH, concentration/protein expression, and post-translational modifications, as the condensate always becomes more similar to the dilute phase when approaching the critical point.

As stated before, condensates in cells are believed to exist close to their critical points to allow the cell to actively control their formation and dissolution.^33^ Our observations indicate that close to the critical point, the changes in condensate volume by fluctuations in environmental conditions or post-translational modifications are nontrivial and might result in either shrinkage or growth of droplets, depending on how close to the critical point the condensates are. Such disparate changes in condensate volume were also observed by Li et al. in response to compression of cell volume.^63^

## 4 Conclusions

Taken together, our results show that studying the volume fraction of condensates can provide fundamental insights into phase separation. Salt-induced dissolution of condensates occurs through a network-expansion and a dissolution regime, and the width of these regimes is determined by the degree of cross-linking in the condensate, which depends on both the length and charge density of the condensate components and on the compositional distance between the condensate and the average concentration of the mixture. More generally, destabilization of biomolecular condensates in the cell could also result in either condensate swelling or shrinkage, a mechanism that could be exploited by cells to regulate condensate volumes.^32^

## Supporting Information Available

Additional details and figures of methods for volume determination and supplementary figures of evolution of volume fraction as a function of salt for different condensate systems (PDF)
