# Supplementary material for: Quantification of Biomolecular Condensate Volume Reveals Network Swelling and Dissolution Regimes during Phase Transition
Source: Biomacromolecules. 2024 Dec 2;26(1):363–73. doi: 10.1021/acs.biomac.4c01201 (PMC11733949; doi:10.1021/acs.biomac.4c01201)
Supplement: Supplementary file 1 — bm4c01201_si_001.pdf [file bm4c01201_si_001.pdf]

Supplementary Information

Quantification of biomolecular condensate volume reveals network swelling and dissolution regimes during phase transition

Iris B. A. Smokers[a], Evan Spruijt*[a]

[a] Institute for Molecules and Materials, Radboud University, Heyendaalseweg 135, 6523 AJ Nijmegen, The Netherlands. E-mail: e.spruijt@science.ru.nl

Contents

1. Supplementary description of volume determination methods ... 3
1.1. 3D-confocal: microfluidics ... 3
1.2. 3D-confocal: Settled condensates ... 3
1.3. Calibrated fluorescence intensity ... 3
1.4. Calibrated micropipette ... 4
1.5. Mass-based method (mass fraction) ... 4
1.6. Calibrated height measurement ... 4
1.7. Cell counting tubes ... 4
1.8. Sessile droplet ... 4
2. Supplementary experimental procedures ... 4
2.1. Condensate preparation: PMETAC / Fl-PSPMA ... 4
3. Supplementary figures and procedures for volume determination methods ... 5
3.1. Mass-based method ... 5
3.2. Water content determination ... 6
3.3. Calibrated height measurement ... 6
3.4. Cell counting tubes ... 8
3.5. Sessile droplet method ... 8
4) Supplementary data ... 10
5) Data repository ... 12
6) Supplementary references ... 12

1. Supplementary description of volume determination methods

1.1. 3D-confocal: microfluidics

Condensate volume can be measured reliably in water-in-oil droplets or vesicles prepared by microfluidics, but does require effort to set up.[1–3] The condensate components can be flown in from two separate microfluidic channels to form condensates in situ. The mixed aqueous solution can then be pinched off into individual droplets and encapsulated in an oil phase. By setting the right flow rates, both the mixing ratio of the condensate components and the size of the water-in-oil droplets can be controlled. Inside the water-in-oil droplets, condensate droplets will nucleate and coalesce over time into a single condensate droplet suspended in the water droplet. The volume fraction of the condensate phase can be determined by taking a z-stack of the entire water-in-oil droplet by 3D-confocal microscopy and determining the maximum radius of both the condensate droplet and the water-in-oil droplet, assuming both are spherical.[3] The advantage of this system is that it only requires very small volumes of 20 – 50 pL per droplet (although larger volumes overall) and that it is high-throughput. Many water-in-oil droplets with different compositions of the condensate components can be prepared by changing the mixing ratios of the components. A disadvantage is that fluorinated oils or surfactants are required to stabilize the water-in-oil droplets, and proteins may denature when they come into contact with these compounds. In addition, the flow rates have to be calibrated well to ensure that the desired mixing ratio is accurately obtained inside the water-in-oil droplets.

1.2. 3D-confocal: Settled condensates

A second method to determine the condensate volume fraction by confocal microscopy is to measure a large z-stack of fluorescently labelled condensates that have settled on a passivated microscopy slide. Condensates are prepared (10 – 100 µL) and added to a microscopy slide and should be given time to settle (while avoiding evaporation of the sample). To get a representative picture of the entire system, a large z-stack should be measured that includes all of the dilute phase. To this end, Peeples et al. used a 200 µm z-stack to get an image that was representative of the whole sample.[4] Although this method has the advantage that it allows for volume determination *in situ*, such large z-stacks are prone to optical aberrations, making the measurement less reliable. In addition, an appropriate surface modification has to be found so that the droplets remain spherical and do not wet the surface of the slide.

1.3. Calibrated fluorescence intensity

The volume fraction of settled condensates can also be measured by making use of the conservation of mass. Fluorescent guest molecules will partition between the condensate phase and the dilute phase,

and therefore, if the total mass of guest in the sample and the respective concentrations in the condensate and dilute phase are known, the volume fraction of the condensates can be determined.[4] The concentration in the condensate phase and dilute phase can be determined by regular confocal fluorescence microscopy using a calibration curve of the fluorescence intensity versus the concentration of the guest molecule. The advantage of this method is that it can be used *in situ* and can therefore easily be combined with other (partitioning) experiments and analyses. The main disadvantages are that differences in quantum yield of the fluorophore between the condensate and dilute phase may give rise to aberrations, and for strongly partitioning guest molecules, the signal in the dilute phase may be too low to quantify accurately. In addition, an appropriate surface modification has to be found so that the droplets remain spherical and do not wet the surface of the slide.

1.4. Calibrated micropipette

Described in detail in main text.

1.5. Mass-based method (mass fraction)

Described in detail in main text, Methods and Supplementary Information Section 3.1.

1.6. Calibrated height measurement

Described in detail in main text, Methods and Supplementary Information Section 3.3.

1.7. Cell counting tubes

Described in detail in main text, Methods and Supplementary Information Section 3.4.

1.8. Sessile droplet

Described in detail in main text, Methods and Supplementary Information Section 3.5.

2. Supplementary experimental procedures

2.1. Condensate preparation: PMETAC / Fl-PSPMA

Charge-neutral condensate emulsions of 50 mM (monomer-based) poly[2-(methacryloyloxy)-ethyl]trimethylammonium chloride (PMETAC, N = 170, PDI = 1.3) and 50 mM (monomer-based) 5% fluorescein-labelled poly(3-sulfopropyl methacrylate) potassium salt (Fl-PSPMA, N = 210, PDI = 1.3) in 100 mM Tris pH 8.5 with 1 M sodium chloride were prepared using stock solutions of 200 mM PMETAC in 100 mM Tris pH 8.5 with 1 M sodium chloride, 200 mM Fl-PSPMA in 100 mM Tris pH 8.5 and 5 M sodium chloride in MilliQ water. Both stock solutions in buffer were corrected back to pH 8.5 using 1 M NaOH and 1 M HCl. For a 1 mL condensate sample, the required volume of 5 M sodium chloride

and 250 µL 200 mM PMETAC were added to the 100 mM Tris pH 8.5 and the solution was mixed by vortexing for a few seconds. Subsequently, 250 µL 200 mM Fl-PSPMA was added, upon which the solution became turbid. The emulsion was mixed by vortexing for a few seconds and inverting the tube at least 3x.

3. Supplementary figures and procedures for volume determination methods

3.1. Mass-based method

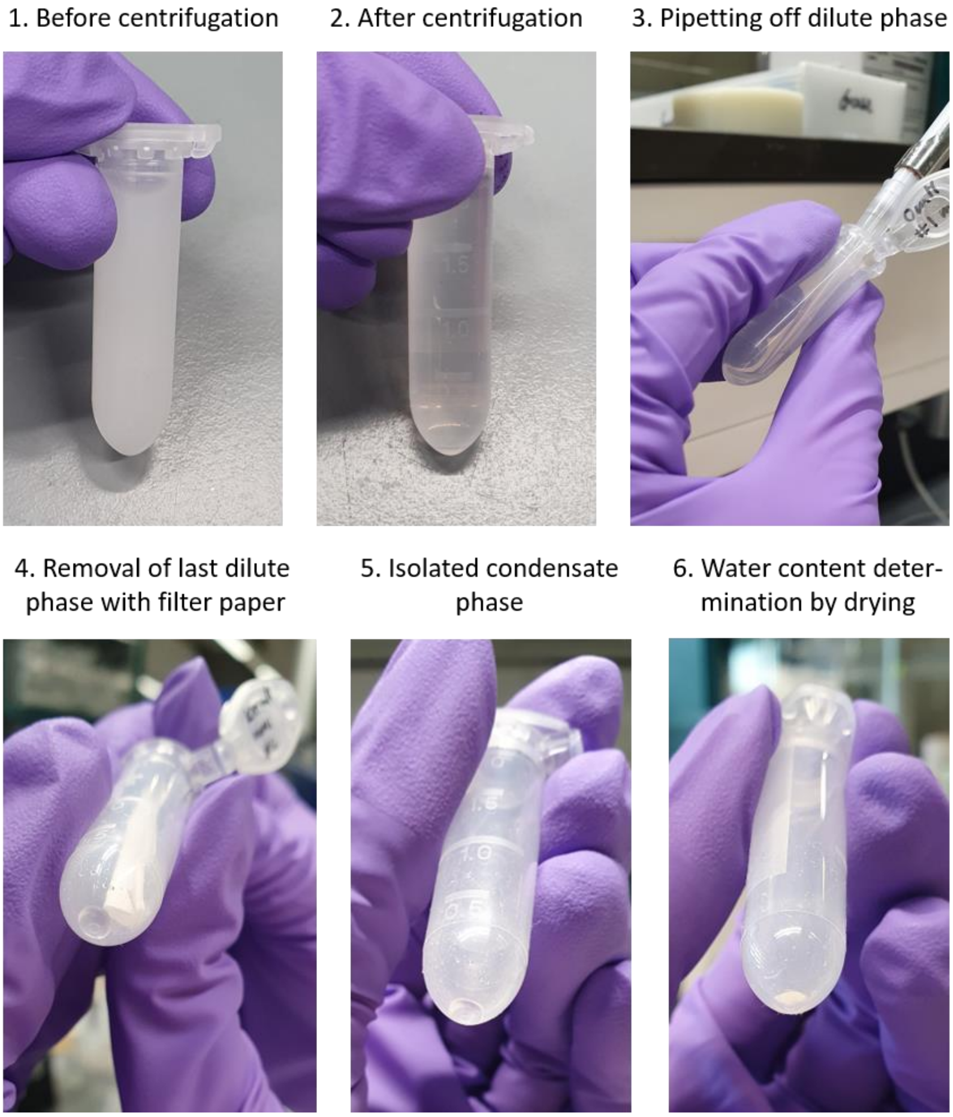

Supplementary Figure 1: Determination of the condensate mass fraction for 1 mM protamine / 25 mM ATP in 100 mM Tris pH 8.5, showing the sample before and after centrifugation. After centrifugation the bulk dilute phase is removed by micropipette after which the last bit of dilute phase is removed from the condensate phase using filter paper. The resulting isolated condensate phase is weighed and can be dried at 120 ˚C to determine the water content.

3.2. Water content determination

After isolating the condensate phase using the procedure of the 'Mass-based method', the tube with condensate phase can be weighed and subsequently placed without lid in a vacuum oven, where it is dried at 120˚C for 48 hours. The dried condensate becomes a transparent solid with cracks. After cooling to room temperature, the sample should be weighed again and the water content can be calculated according to Equation 1:

$$Water\ content = \frac{m_{\mathrm{cond}} - m_{\mathrm{dry}}}{m_{\mathrm{cond}} - m_{\mathrm{empty}}} \qquad \textit{Supplementary Equation 1}$$

3.3. Calibrated height measurement

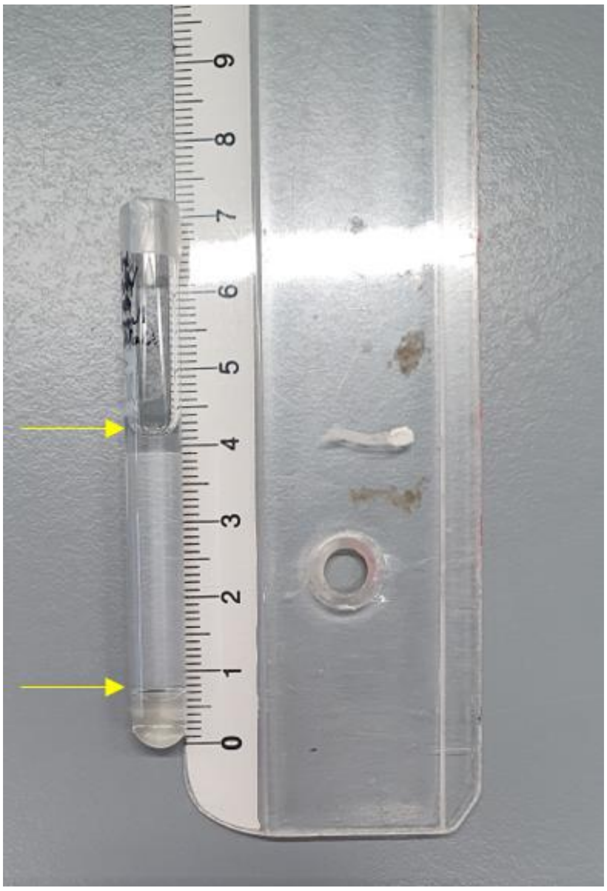

Supplementary Figure 2: Condensate volume read-out with a ruler to measure the height of the condensate phase and total sample. The line in the sample at 3.9 mm is a reflection of the light from the interface.

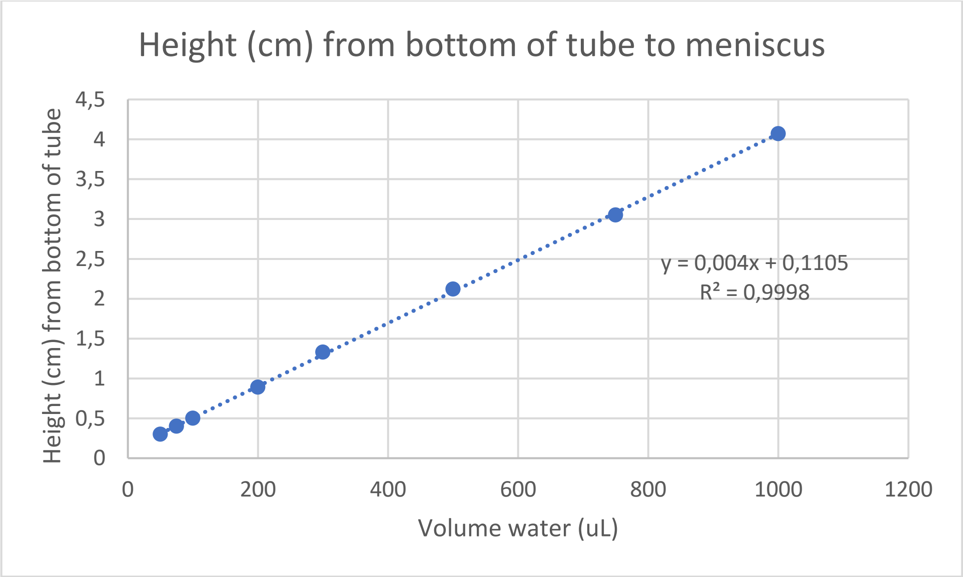

Supplementary Figure 3: Calibration curve for volume determination by calibrated height measurement in narrow test tubes, prepared using known volumes of MilliQ water, which were centrifuged for 1 minute at 3095 RCF and 20°C.

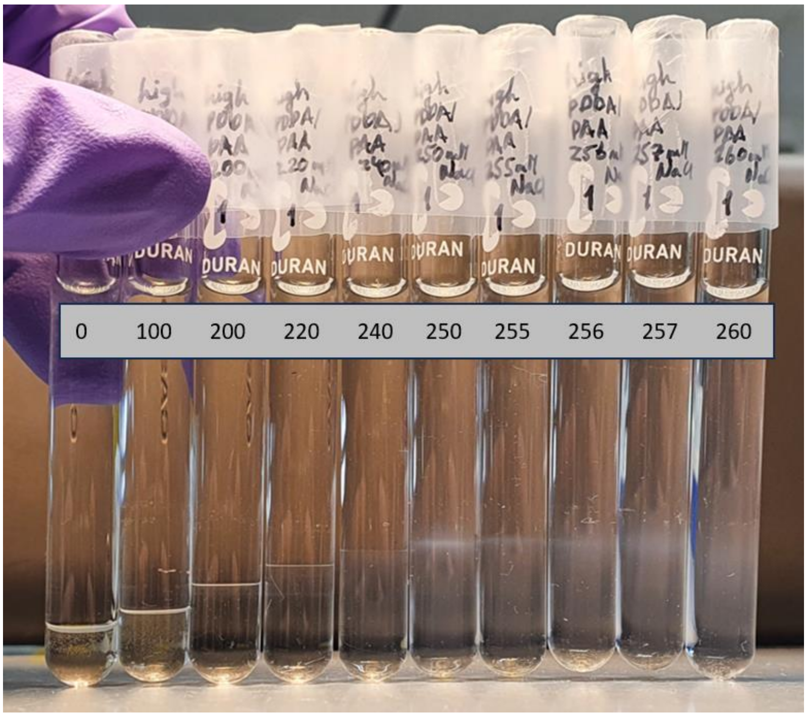

Supplementary Figure 4: Narrow tubes with 150 mM PDDA/PAA condensates with different concentrations of sodium chloride (concentrations in mM). The phases become more similar in refractive index, and the interface becomes more diffuse for higher salt concentrations and required a short heat-shock to be visualized.

3.4. Cell counting tubes

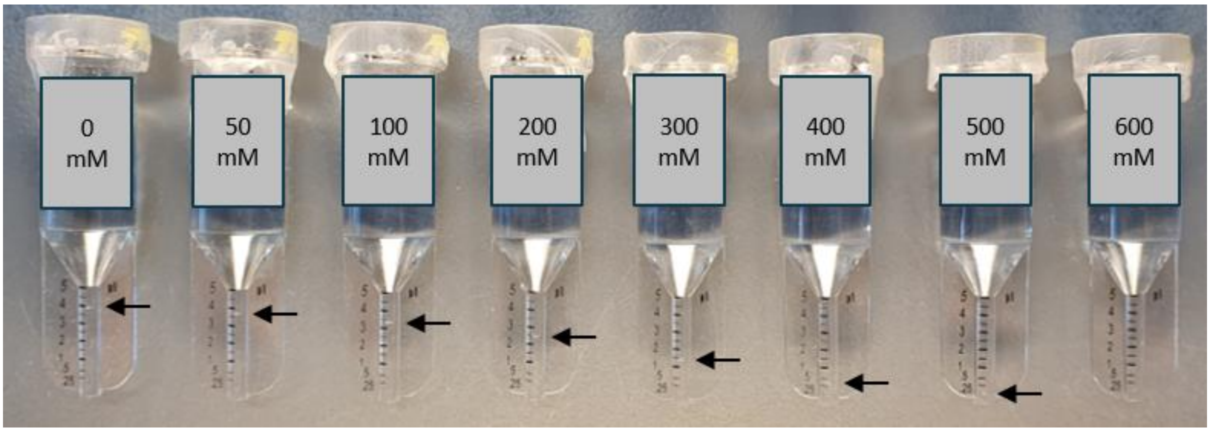

Supplementary Figure 5: Read-out of the total condensate phase volume from cell counting tubes for 1 mM protamine / 25 mM ATP condensates with different concentrations of sodium chloride.

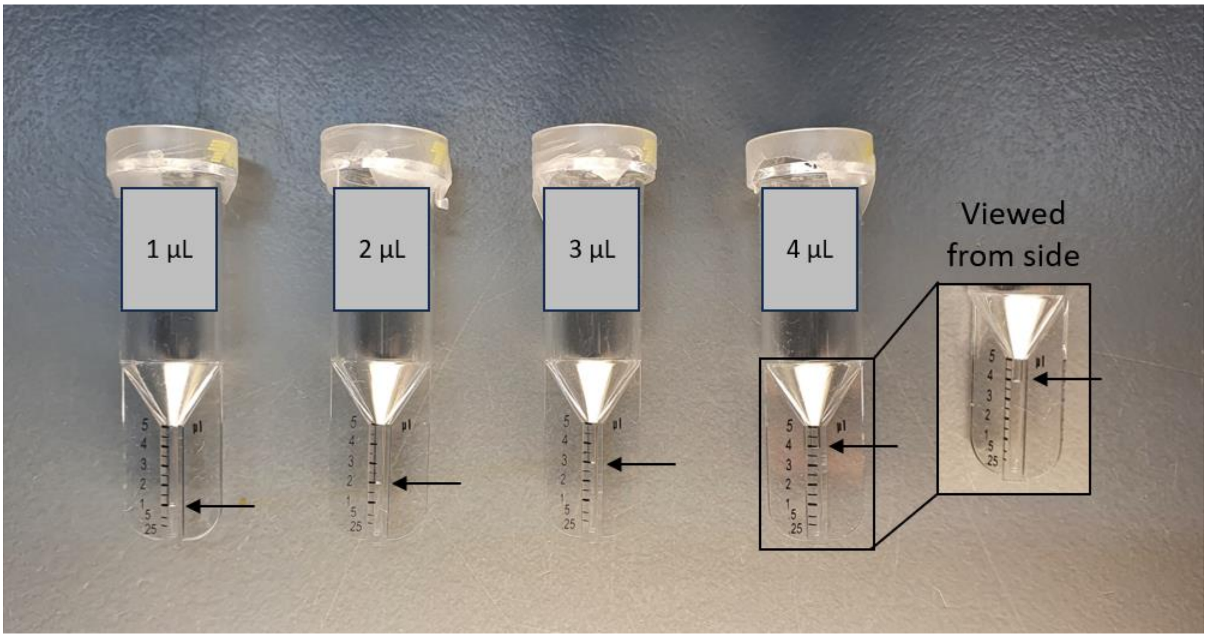

Supplementary Figure 6: Control for cell counting tube read-out. 1, 2, 3 or 4 µL MQ-water was added to cell counting tubes, after which they were centrifuged for 30 seconds.

3.5. Sessile droplet method

The condensate droplet volume was calculated from the obtained images using *ImageJ*. Using the known dimensions of the cuvette chamber (2 mm width), the dimensions of the droplet could be calculated using the spherical cap method (Supplementary Figure 7, left). Dimensions *2a* and *h* were determined in ImageJ according to the procedure in Supplementary Figure 8, after which the droplet volume could be calculated using Equation 2:

$$V = \frac{1}{6}\pi h(3a^2 + h^2) \qquad \textit{Supplementary Equation 2}$$

For less spherical droplets ($2a$ / h > 1.5), alternatively the elliptical cap formula can be used (Supplementary Figure 7, right). Dimensions h and $2b$ and d can be determined in ImageJ, and c can be calculated using $c = 2d - h$. The droplet volume can then be calculated using Equation 3:

$$V = \frac{\pi b}{3d^2} \cdot (4d^3 + c^3 - 3c^2 d) \qquad \textit{Supplementary Equation 3}$$

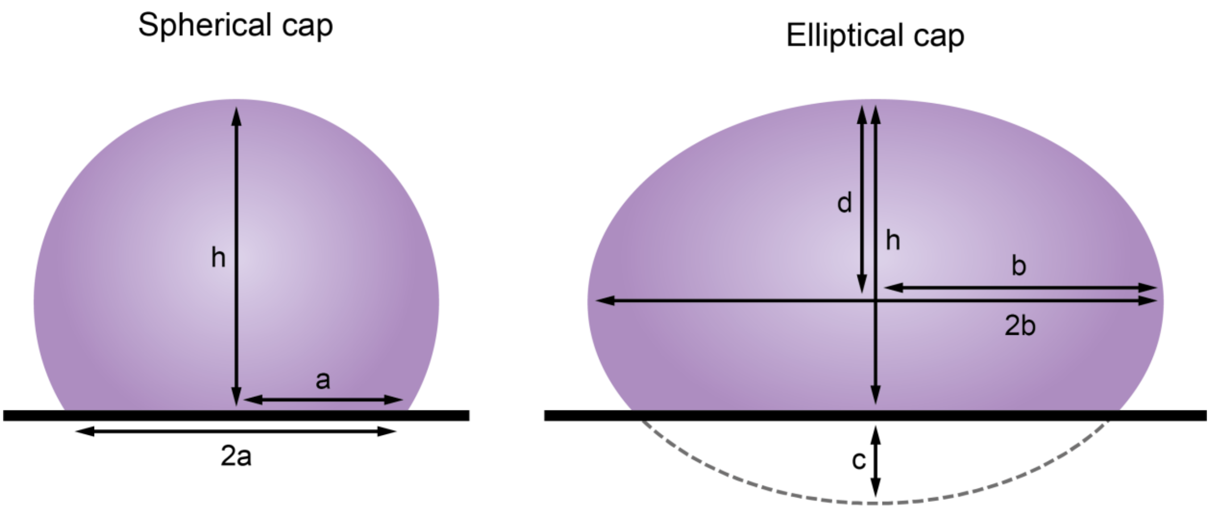

Supplementary Figure 7: Dimensions required for calculating the droplet volume using the spherical cap (left) and elliptical cap (right) method.

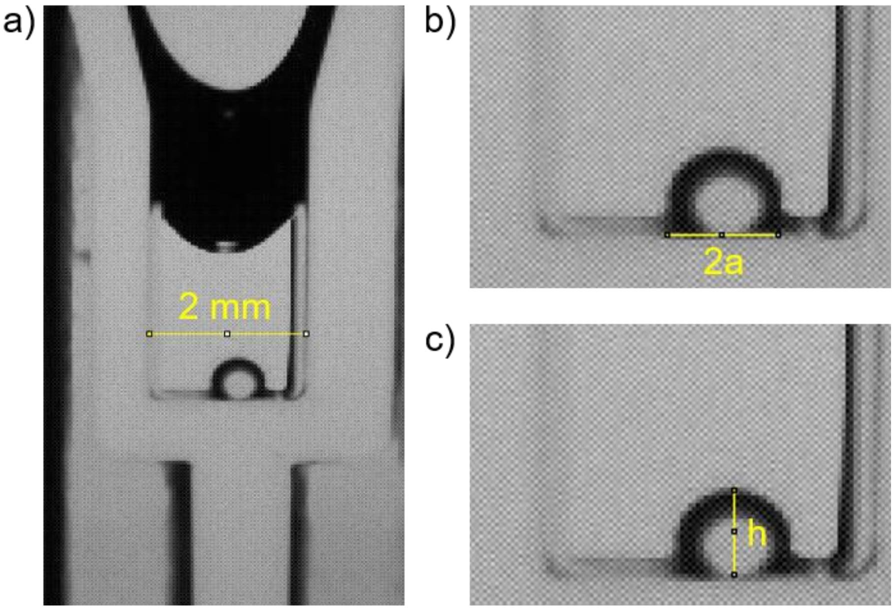

Supplementary Figure 8: Measurements for the spherical cap method in ImageJ. a) The known width of the cuvette chamber is used to set the image scale. After which the dimensions of the droplet are determined: b) the width *2a* of the droplet where it touches the cuvette surface & c) the height *h* of the droplet.

4) Supplementary data

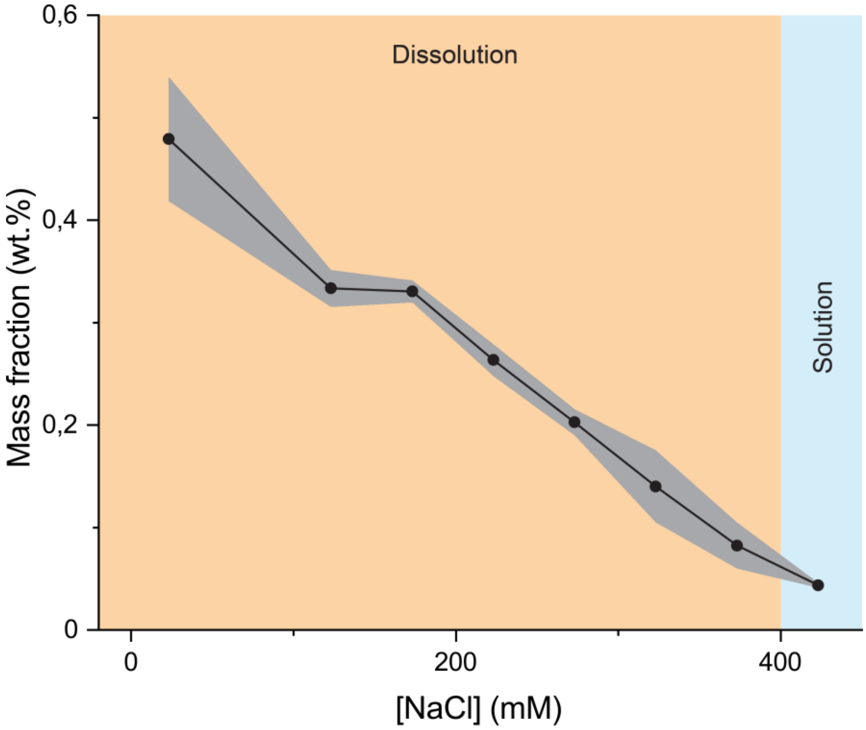

Supplementary Figure 9: Condensate mass fraction of 1 mM protamine / 25 mM ATP condensates in 100 mM Tris pH 8.5 as a function of total sodium chloride concentration. The mass fraction was determined according to the procedure in the methods section 'Volume determination by mass'. Error bars are shaded in grey and depict the standard deviation of measurements in triplo.

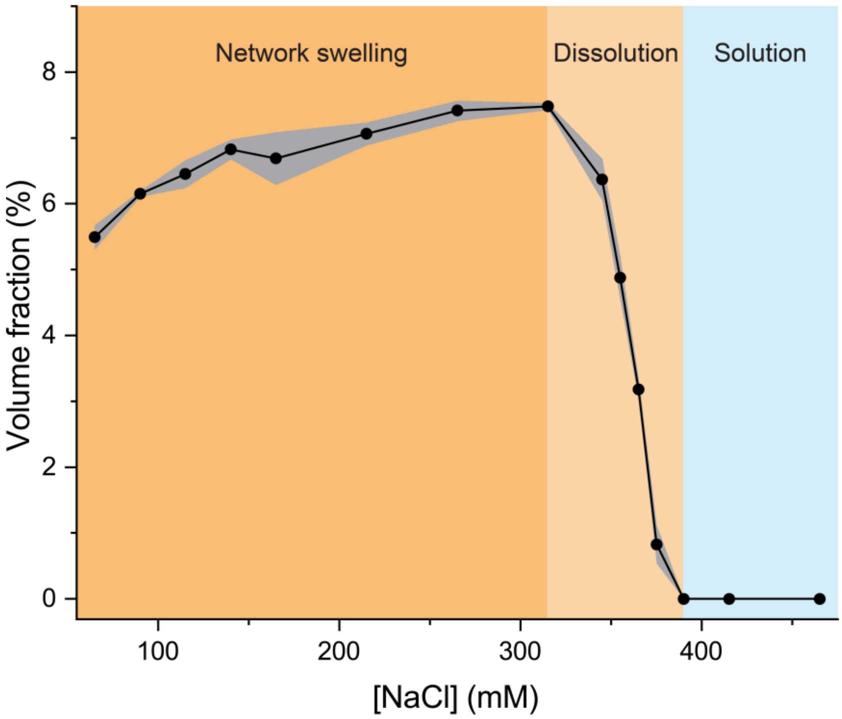

Supplementary Figure 10: Volume of 65 mM PDDA/PAA as a function of total salt concentration measured after equilibrating for one month. Empty triangles indicate the samples where no phase separation was observed. Error bars are shaded in grey and depict the standard deviation of measurements in triplo.

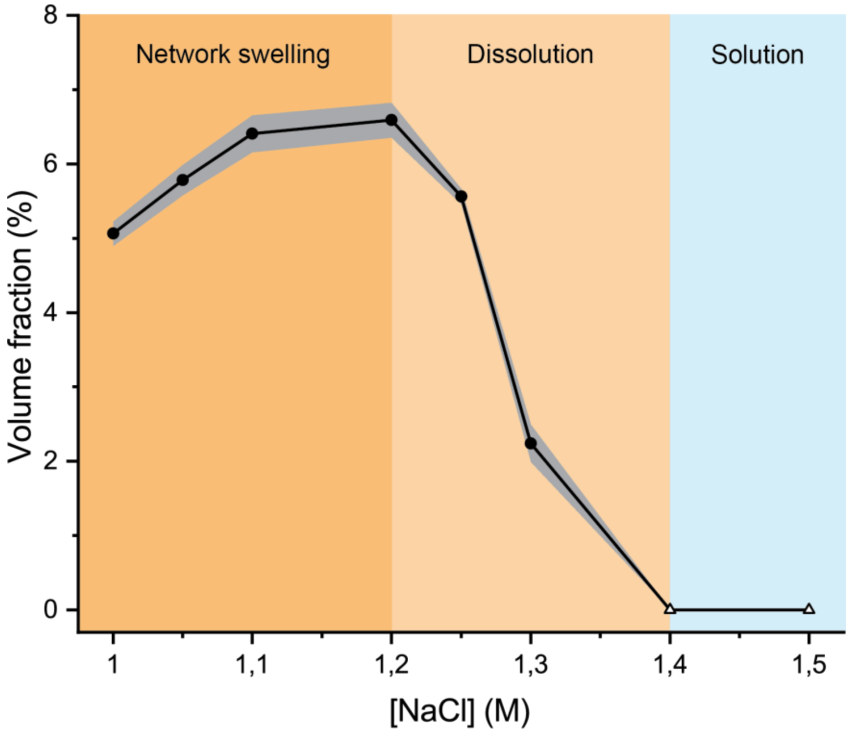

Supplementary Figure 11: Volume of 50 mM PMETAC/(5% fluorescein)-PSPMA as a function of added salt concentration measured directly after centrifugation. In the network-swelling regime, these condensates had a typical relaxation time longer than the timescale of mixing causing them to look gel-like. On the time-scale of an hour they were liquid. Empty triangles indicate the samples where no phase separation was observed. Error bars are shaded in grey and depict the standard deviation of measurements in triplo.

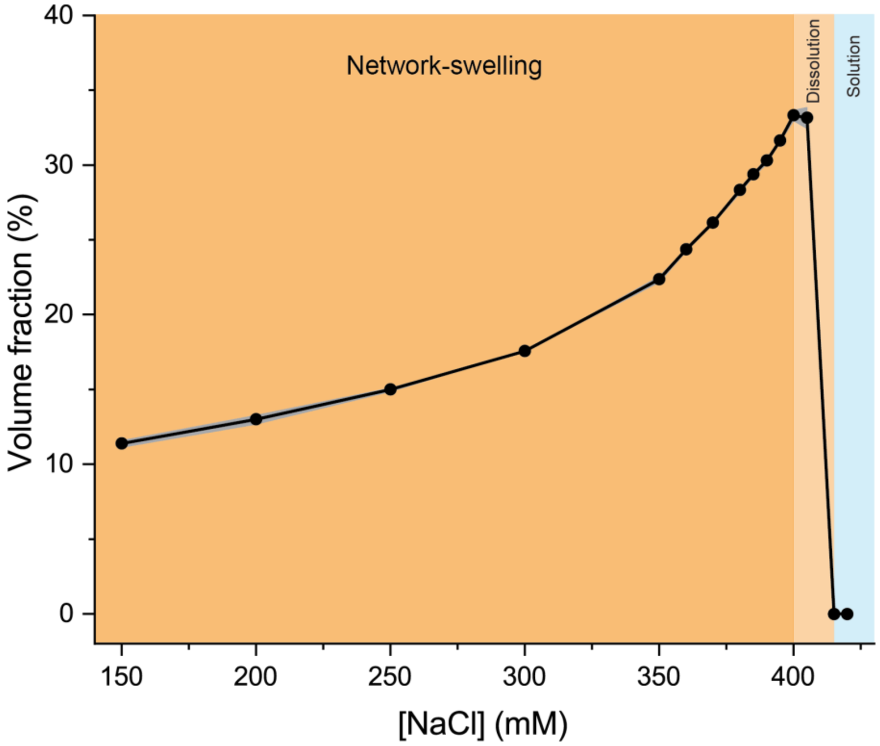

Supplementary Figure 12: Volume of 150 mM PDDA/PAA as a function of total salt concentration measured directly after centrifugation. Empty triangles indicate the samples where no phase separation was observed. Error bars are shaded in grey and depict the standard deviation of measurements in triplo.

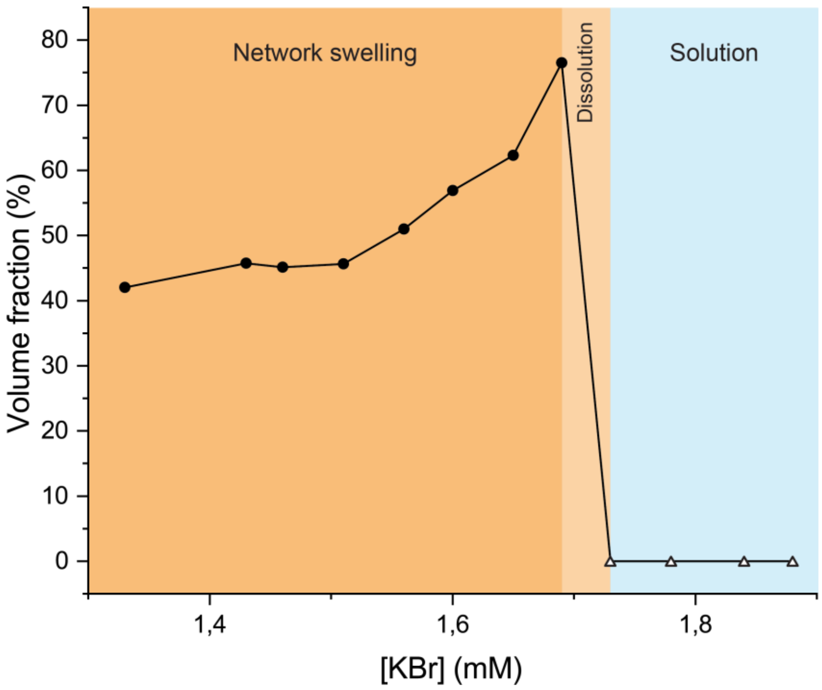

Supplementary Figure 13: Results from Wang & Schlenoff on the volume of 0.1 g/mL PSS/PDADMA condensates as a function of salt concentration measured after letting the samples equilibrate for 30 days.[5] Empty triangles indicate the samples where no phase separation was observed.

5) Data repository

Raw data can be found on the Radboud Data Repository under https://doi.org/10.34973/vf0v-8s11.

6) Supplementary references

(1) Love, C.; Steinkühler, J.; Gonzales, D. T.; Yandrapalli, N.; Robinson, T.; Dimova, R.; Tang, T. -Y. D. Reversible PH-Responsive Coacervate Formation in Lipid Vesicles Activates Dormant Enzymatic Reactions. *Angew. Chemie* **2020**, *132* (15), 6006–6013. https://doi.org/10.1002/ange.201914893.

(2) Beneyton, T.; Love, C.; Girault, M.; Tang, T. -Y. D.; Baret, J. High-Throughput Synthesis and Screening of Functional Coacervates Using Microfluidics. *ChemSystemsChem* **2020**, *2* (6), e2000022. https://doi.org/10.1002/syst.202000022.

(3) Chen, F.; Li, X.; Guo, W.; Wang, Y.; Guo, M.; Shum, H. C. Size Scaling of Condensates in Multicomponent Phase Separation. *J. Am. Chem. Soc.* **2024**. https://doi.org/10.1021/JACS.4C02906.

(4) Peeples, W.; Rosen, M. K. Mechanistic Dissection of Increased Enzymatic Rate in a Phase-Separated Compartment. *Nat. Chem. Biol.* **2021**, *17* (6), 693–702. https://doi.org/10.1038/s41589-021-00801-x.

(5) Wang, Q.; Schlenoff, J. B. The Polyelectrolyte Complex/Coacervate Continuum. *Macromolecules* **2014**, *47* (9), 3108–3116. https://doi.org/10.1021/ma500500q.
